# Off the Beaten Path: Drug Addiction and the Pontine Laterodorsal Tegmentum

**DOI:** 10.1155/2013/604847

**Published:** 2013-06-23

**Authors:** Kristi A. Kohlmeier

**Affiliations:** Department of Drug Design and Pharmacology, Faculty of Health Sciences, Universitetsparken 2, University of Copenhagen, 2100 Copenhagen, Denmark

## Abstract

Drug addiction is a multileveled behavior controlled by interactions among many diverse neuronal groups involving several neurotransmitter systems. The involvement of brainstem-sourced, cholinergic neurotransmission in the development of addiction and in the persistent physiological processes that drive this maladaptive behavior has not been widely investigated. The major cholinergic input to neurons in the midbrain which are instrumental in assessment of reward and assignment of salience to stimuli, including drugs of abuse, sources from acetylcholine- (ACh-) containing pontine neurons of the laterodorsal tegmentum (LDT). Excitatory LDT input, likely cholinergic, is critical in allowing behaviorally relevant neuronal firing patterns within midbrain reward circuitry. Via this control, the LDT is positioned to be importantly involved in development of compulsive, addictive patterns of behavior. The goal of this review is to present the anatomical, physiological, and behavioral evidence suggesting a role of the LDT in the neurobiology underlying addiction to drugs of abuse. Although focus is directed on the evidence supporting a vital participation of the cholinergic neurons of the LDT, data indicating a contribution of noncholinergic LDT neurons to processes underlying addiction are also reviewed. While sparse, available information of actions of drugs of abuse on LDT cells and the output of these neurons as well as their influence on addiction-related behavior are also presented. Taken together, data from studies presented in this review strongly support the position that the LDT is a major player in the neurobiology of drug addiction. Accordingly, the LDT may serve as a future treatment target for efficacious pharmaceutical combat of drug addiction.

## 1. Introduction

Drug addiction is a complex, maladaptive behavior that relies certainly on a tangled interplay of different cellular and network level physiological processes. Processes underlying learning and memory, experience of euphoria, reward, physical withdrawal, depression, and arousal are only some of those involved in manifestation of drug use, abuse, and the dependence cycle. These processes probably do not operate in isolation but are likely altered by interactions at the cellular level within responsible neuronal centers, making deconstruction and examination of their individual role in the development and maintenance of addiction difficult. Certainly, the neural processes mediating experience of euphoria and evaluation of the reward associated with a stimulus are integral to the development of drug dependence. Therefore, focus on the cellular mechanisms underlying the neurobiology of addiction to drugs of abuse has reasonably been centered on the midbrain dopamine- (DA-) containing neurons of the ventral tegmental area (VTA) as it is clear from anatomical, physiological, and behavioral studies that these neurons play a vital role in the neurocircuitry critical for processes underlying the experience of euphoria and reward and, accordingly, development of addiction. The VTA consists of DA- and GABA-containing projection neurons, as well as local GABA-containing cells. The VTA is highly segregated, exhibiting multiple connectivity pathways. There is a high degree of specificity existing in VTA afferents to target regions as distinct populations of cells project to different target fields [1]. Such specificity in connectivity is indicative of distinct subregions subserving different functions. The VTA is divided into distinct subregions or subnuclei, designated in some studies as anterior, posterior, tail, lateral, or ventral, depending on the plane of orientation utilized [2–6]. DA cells within distinct regions of the VTA respond differentially to drugs of abuse, with different input and output projection patterns likely involved [5–11]. Recently, an electrophysiological study showed that posterior DA VTA neurons were more strongly excited by nicotine when compared to membrane actions elicited by this addictive drug on neurons in the anterior or tail of the VTA [12]. These data suggested a greater role played by posterior VTA neurons in neural processing leading to assignment of a positive valence to nicotine [12]. DA neurons in the ventromedial VTA were found to be excited by lack of stimuli associated with prediction of a reward, suggesting a role of DA neurons within this anatomical region in processing of stimuli with low motivational value [13]. The tail of the VTA, which is also defined as the rostromedial tegmental nucleus, exerts an inhibitory drive on DA release and is therefore poised to participate in evaluation of saliency of behaviorally relevant stimuli [14]. Opioids are believed to excite DA VTA neurons via disinhibition, which incorporates an opioid-mediated silencing of GABAergic VTA cells. A rationale for refinement of this model suggests that as GABA neurons within the tail of the VTA express high levels of *μ*-opioid receptors, and these cells send a heavy projection to DA VTA neurons; data from earlier studies leading to the development of the opioid disinhibition model may have included recordings from VTA tail neurons [14]. The findings from studies that suggest differences in the responsitivity of DA VTA neurons across anatomical subregions are clearly of behavioral importance, and greater care should perhaps be taken to distinguish among them in cellular studies of the role of the VTA in physiology and behavior. However, while that caveat must be kept in consideration, most studies of VTA neuronal functioning to date have not compared data between regions. 

Although the VTA sends efferent projections to a diversity of neural structures, the extent of which was not fully recognized initially [3, 15]; the projections to the medial prefrontal cortex (PFC-mesoprefrontal pathway) and nucleus accumbuns (nAcc-mesoaccumbal pathway) have been implicated to play a vital role in the neurophysiological pathways important in mediating activity involved in the development of addiction to drugs of abuse. A common feature of drugs that shows abuse potential is their ability to substantially enhance DA levels within the mesoaccumbal pathway associated with induction of a burst firing pattern in DA VTA cells. Upon exposure to drugs of abuse, appreciably higher levels of DA can be measured within the nAcc, preceded by excitation of DA-containing VTA neurons. Although most studies of this drug-stimulated effect have been conducted using animal models, euphoric actions of stimulant drugs in humans have been found to correlate with levels of DA within the ventral striatum, which includes the nAcc, with greater self-reported hedonic experiences correlated with higher DA levels [16]. A compendium of findings from addiction studies now indicate that firing of DA VTA neurons signals a reward prediction error as well as the salience of a stimulus or its novelty [17, 18], with cortical neurons perhaps discriminating a reward [18–22]. This is a finer distinction of earlier, more simplistic views that release of DA from the VTA signaled reward. The redefinition of the role played by the mesoaccumbal pathway in evaluation of environmental stimuli was necessitated, in part, by findings that negative stimuli, such as stress, also activate DA VTA neurons presumably within the same VTA subregion [23]. The stress-mediated activation shares cellular mechanisms of activation of these cells by several addictive drugs, suggesting that not only do positive stimuli serve to activate the VTA, but also that negative stimuli are capable of similar activation [23]. Mechanisms by which drugs of abuse activate DA VTA neurons vary and are complex and can depend upon interactions of these drugs with non-DA-containing VTA neurons, cooccurring with or in absence of, direct actions on DA VTA cells. In addition, since actions of drugs of abuse are not uniquely targeted to the VTA, direct and indirect actions of drugs of abuse on neurons of the VTA are not the sole drivers of activity of DA VTA cells. Accessory neuronal regions and neurotransmitters, the activity of which may be altered by drugs of abuse, must necessarily be also involved. 

Examinations beyond the VTA have identified a role in addiction played by the laterodorsal tegmentum (LDT), and specifically, that cholinergic neurons of the LDT may play a vital role in addiction processes via participation in two relevant afferent pathways. Most studies of the role played by cholinergic LDT neurons in behavior have focused on their control of behavioral arousal. Cholinergic neurons of the LDT were recognized to contribute significantly to arousal as critical players within the reticular activating system (RAS). The RAS provides the cholinergic input from the mesopontine region to the thalamus and the basal forebrain, influencing more rostral cortical centers [24]. Cholinergic activation of the cortical regions by the midbrain cholinergic neurons of the RAS leads to states of high behavioral arousal concurrent with a cholinergic mediated shift of the EEG pattern from one of quiet wakefulness to that associated with high attention [25–31]. While arousal was a well-recognized one process operating during drug-seeking behaviors, a more direct role of the LDT in the neurocircuitry of addiction was not really explored until the last decade when it was discovered that midbrain ACh-containing neurons provide a major cholinergic input to the VTA [32, 33]. It is now believed that these mesopontine cholinergic neurons source as the primary cholinergic input to this region and are therefore the main drivers of activation of cholinoceptive receptors located postsynaptically on DA and non-DA VTA cells [32, 33]. Cholinergic stimulation of nicotinic and muscarinic ACh receptors within the VTA results in excitation of the postsynaptic cell suggesting that the LDT could play a major role in control of DA neuronal excitability. Studies were therefore conducted examining the role that the LDT plays in addiction processes. The data emerging from these studies indicate that cholinergic mechanisms within the VTA, likely sourcing from the LDT, play a critical role in drug addiction-associated behaviors via activation of DA VTA neurons. Burst firing, which is the behaviorally relevant firing pattern of DA VTA neurons and leads to large efflux of DA within the nAcc, relies critically on a functioning LDT. Additionally, the high degree of behavioral arousal during drug addiction characteristic behaviors, such as drug-seeking activity, suggests high activity of the cholinergic neurons of the LDT. The LDT driven arousal may source from activity within rostral projections driving control of cortical centers, and this mesopontine driven arousal is likely importantly involved in the physiology driving this maladaptive, complex behavior. This review examines the available literature implicating activity of cholinergic neurons of the LDT in drug addiction-related physiology and behaviors. Also included is a discussion of recently reported findings compellingly suggesting a role played in addiction processes by noncholinergic neurons of the LDT. Taken together, the studies suggest that one future goal of treatment of this devastating disorder may include pharmaceutical targeting of activity of selective populations of neurons of the LDT. 

## 2. The Anatomy of the LDT

**Figure 1:**
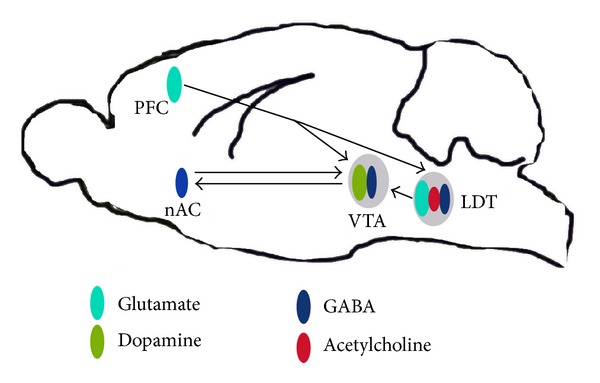
Highly simplified schematic showing relevant anatomical connectivity of the laterodorsal tegmentum (LDT) within known addiction-related pathways in the rat brain. The LDT contains separate populations of cholinergic, GABAergic, and glutamatergic neurons. A subset of these cells sends projections to the ventral tegmental area (VTA) synapsing on GABAergic and dopamine-containing cells in this nucleus which project to the nucleus accumbens (nAC). The prefrontal cortex (PFC) exerts control over the VTA via direct projections to this nucleus and via an indirect pathway synapsing within the LDT.

The LDT is included in the midbrain cholinergic center that is divided into the Ch5 and Ch6 cholinergic cell groups, which are located in the mesopontine tegmentum. The mesopontine tegmentum is divided into the more rostral pedunculopontine tegmentum (PPT, A5) and the more caudal LDT (A6) [27, 28]. Although apparently similar in physiology, function, and transmitter content [34–42], detailed studies have revealed physiological differences of, and divergent projection patterns arising from, the LDT when compared to those of the PPT. Such divergent projections probably underlie differing neurophysiological roles of these two cholinergic nuclei in control of diverse behaviors. Relevant to this review, the PPT largely innervates the substantia nigra [41, 43–46], whereas the LDT, with perhaps a small contribution from the most caudal PPT, provides the major cholinergic innervation of the VTA, as well as a noncholinergic afferent pathway [47]. Given the paramount role of the VTA in development of drug addiction, focus on the role of the mesopontine cholinergic groups in the neurophysiology important in development of addiction and associated behaviors will be directed to the contribution of the LDT. Excellent reviews of the role of the PPT in arousal-related behaviors have been presented and are available for those interested in contributions of this neuronal group to physiological processes and behaviors [48–50]. Within the pontine tegmentum, the dorsal edge of the LDT is positioned next to the fourth aqueduct with its caudal tail laterally adjacent to the locus coeruleus. Although the principal cell type within the LDT is considered to be cholinergic, in fact cholinergic neurons do not represent the predominant cell type. GABA-containing cells outnumber the cholinergic cells within the LDT by approximately two to one [42, 51]. A nonhomogenous distribution of cell phenotype was noted with the highest concentration of ACh neurons present in the medial LDT. Even though the medial LDT contained the largest numbers of cholinergic neurons, the numbers of ACh-containing cells within this region were found to be similar to the numbers of the other two cell phenotypes [51]. The rostral LDT contained the highest percentage of glutamate-containing neurons, while the highest percentage of GABA-containing neurons were present in the caudal LDT [51]. Based on initial studies, it was once thought that GABA could colocalize within at least a subset of the population of ACh-containing LDT cells [37]. However, more recent data has necessitated revision of this thinking, and it is now believed that the GABAergic and cholinergic neurons represent distinct populations [51]. Similarly, evidence had been presented that glutamate colocalized with ACh in the LDT leading to the assumption that glutamate and ACh could be co-released at targets of terminals sourcing from neurons of the LDT [35, 36]. However, more recent findings using mRNA localization of glutamate transporters argue against colocalization of these two neurotransmitters and strongly suggest that ACh- and glutamate-containing cells represent distinct populations within the LDT [51]. Outflow from the LDT to target regions is probably dependent on an intricate counterbalance of activity within these phenotypically distinct cell groups, as well as influences directed to LDT cells from projections arising outside the nucleus (Figure 1).

## 3. Connectivity of the LDT: The LDT-VTA Pathway

Projections from the LDT have been studied, and the presence of synaptic connectivity between the LDT and the VTA has been established including determination of the transmitter content of target cells receiving synaptic contact within the VTA from the LDT. First reports suggested that ACh cells targeted VTA neurons with a very low content of DA, which suggested LDT innervation predominantly of mesoprefrontal DA neurons and sparse innervation of neurons comprising the mesoaccumbal pathway [52]. However, refuting the earlier reports, it was determined that ACh-containing synaptic contacts were preferentially directed to mesoaccumbal DA-containing VTA cells when compared to numbers of synapses made onto mesoprefrontal DA neurons or GABA cells within either pathway [32]. Based on the asymmetric profile of these synapses, these contacts were likely to be excitatory [32]. It was not possible within that study to definitively establish that the asymmetric synapses directed to mesoaccumbal DA VTA neurons sourced from the LDT. However, when the profiles of these contacts were compared to that seen in an earlier study examining the innervation of the VTA deriving from the LDT, it was concluded that a substantial proportion of the contacts likely derived from ACh-containing LDT neurons, although a contribution of cholinergic synapses on DA VTA neurons deriving from other nuclei could not be ruled out [32, 33]. In addition, excitatory, noncholinergic input from the LDT was also indicated. Identification of synaptic sources on DA VTA cells positioned the cholinergic afferent input from the LDT as likely to play a role in DA-involved processing of reward and development of motivation underlying drug abuse. Whether a distinct subset of LDT neurons among the cholinergic population sends projections to the VTA remain to be determined. Cholinergic neurons have been shown to innervate multiple target structures through divergent axonal branches [24, 53–57]. However, further studies have revealed that some targets are innervated by separate populations of cholinergic LDT cells. Cholinergic axon terminals deriving from the LDT were found to be different depending on whether they were directed to the anteroventral thalamic nucleus or to the VTA, and data suggested copresence of neuropeptides and ACh in synapses terminating within the VTA [58]. Further experiments utilizing dual retrograde tract tracing methodology showed that only a small percentage of cholinergic LDT neurons collateralize to both nuclei, with the majority of cholinergic neurons sending projections to one nucleus or another [59]. As it is possible that an as yet unidentified select population of LDT neurons project to the VTA, it would be of interest to determine whether this subpopulation exhibits a distinctive firing pattern that could underlie their control of the VTA DA cells. Also, it is unknown whether firing of VTA-projecting cholinergic LDT neurons is distinguishable from firing that is shown to be critical in control of another process in which a high degree of cholinergic activity within the LDT plays a role, such as in induction and maintenance of the phase of sleep called rapid eye movement (REM) sleep. Generation and maintenance of REM sleep are reliant upon firing of cholinergic neurons and cholinergic activation of relevant target regions located both rostrally and caudally [39, 40, 60]. While single cholinergic neurons can innervate thalamic and caudal regions and are therefore likely to be importantly involved in generation of REM sleep [24, 57], it is currently unknown if these putatively REM-involved cells represent a different population of those sending terminals to the VTA. Interestingly, stimulus-induced selective burst firing of the population of cholinergic neurons projecting to the VTA could result in release of peptides [61, 62] demonstrated to be colocalized in cholinergic LDT neurons [58, 63] and suggested to be present in projections directed to the VTA [58]. Peptide actions within the VTA, combined with a high efflux of ACh, could play a role in DA-mediated assignment of reward to drugs of abuse via LDT driven high DA VTA cell firing. Further studies should clarify the identity and firing behavior of the cholinergic LDT cells which target the LDT.

## 4. Functional Activation of the LDT-VTA Pathway

The anatomical pathway from the LDT to the VTA can be functionally activated. Use of *in vivo* chronoamperometry and microdialysis showed that stimulation of the LDT in anesthetized rats results in a complex efflux of DA in mesoaccumbal target regions of the VTA, which was dependent upon three different cellular mechanisms [64–66]. The first component was stimulatory of DA release and mediated by glutamate and ACh release with the latter acting on nicotinic acetylcholine receptors (nAChRs) on cells within the VTA. The second component was inhibitory to DA release and relied on autoinhibition of cholinergic afferents via activation of inhibitory muscarinic receptors. The third component was a long-lived DA efflux mediated by excitation of M5 type muscarinic ACh receptors on VTA cells. Interestingly, the M5 type muscarinic receptor is the only one of the five known muscarinic receptor subtypes localized on DA VTA neurons, and studies have revealed that this receptor is importantly involved in DA VTA cell functioning controlled by the LDT [64, 66–69]. Pharmacologic inhibition of VTA M5 receptors via application of a muscarinic receptor antagonist reduced both LDT stimulation-induced rises in DA within the nAcc, as well as cocaine-mediated facilitation of this response [70]. Mice deficient in the M5 receptor exhibit smaller rises in DA in the nAcc following LDT electrical stimulation [65]. Animals in which the LDT had been chemically lesioned failed to show a heightened DA release in the nAcc induced by application of acetylcholinesterase inhibitors, suggesting that the LDT provides the endogenous cholinergic input driving ACh activation of the DA VTA cells [71]. Chemical stimulation of the LDT also resulted in efflux of DA within this pathway [72]. 

More novel ways to elicit cellular excitation have also demonstrated functional activation of the LDT-VTA pathway. Optogenetics methodology involves transfection of cells of interest with cation channels that can be gated by pulses of light. When LDT cells, which contribute to the LDT-VTA pathway, were transfected with the cation channel, channelrhodopsin-2, the opening of this channel could be triggered by light pulses directed to transfected cells. Light pulses sufficient to open this channel and activate LDT neurons resulted in high expression levels of cFos in neurons of the VTA, indicating functional connectivity [73]. Further characterization of the input directed to the VTA from the LDT was conducted using a retrograde virus coupled with a fluorescent marker [73]. Following injection of the virus into the VTA, fluorescent cells within the LDT that sent projections to the VTA were identified, and markers for the glutamate transporter and the choline acetyltransferase (ChAT) enzyme were utilized to phenotypically characterize identified afferents [73]. An overwhelming percentage of those VTA-projecting LDT neurons were identified as glutamatergic with only 7% expressing ChAT. Further, use of an anterograde marker revealed that the majority of LDT afferents were directed to the lateral VTA, which heavily innervates the nAcc with only a light innervation of the medial VTA. Afferents were found to be located near DA processes as identified by presence of tyrosine hydroxylase [73]. The glutamate projection sourcing from the LDT was likely functional, as stimulation of the LDT fibers generated excitatory currents in DA neurons that were blocked by an AMPA glutamate receptor antagonist [73]. The majority of DA cells activated by LDT stimulation were part of the mesoaccumbal pathway as they were found to project to the lateral shell of the nAcc, with a less dense projection directed to the medial shell or substantial nigra. Data from this study, taken together with the finding that excitatory synapses abut mesoaccumbal DA neurons [32], some of which probably were glutamate containing, indicates a heavy excitatory amino acid innervation of the VTA from the LDT, and specifically that glutamate-containing input is most likely directed to DA neurons. Determination of the firing of DA VTA cells in response to glutamate stimulation sourcing from the LDT was not possible in this study. The cholinergic input from the LDT, while identified in the optogenetic physiological study as contributing a small component of afferents to the VTA, was not further examined in that work [73]. Accordingly, at this time, the relative contribution of the glutamate-containing LDT neurons and acetylcholine-containing LDT neurons to behaviorally relevant firing of the DA neurons important in addiction remains to be determined. Studies with more selective lesioning of specific populations of LDT neurons, both in terms of neurochemical content and efferent projection profile to the VTA, need to be conducted to define the exact role played by the cholinergic and glutamatergic LDT cells in the firing of DA neurons leading to release of DA to target regions. In this context, selective phenotype cell lesioning with use of already developed neurotoxins selective for ablation of cholinergic neurons could be utilized [74]. Selective cell lesioning combined with optogenetics could allow light-pulse activation of remaining LDT cells contributing to the LDT-VTA pathway simultaneously with unit recordings within the VTA to determine whether burst firing can be induced in absence of cholinergic neurons. With such studies, it can be further elucidated which population(s) of LDT neurons is critically involved in the firing patterns of DA VTA neurons critical for development of drug addiction-associated behaviors. 

## 5. LDT-Mediated Gating of Behaviorally Relevant Firing Patterns of VTA Neurons

Burst firing of DA VTA neurons has been demonstrated to be a necessary activity pattern for development of neurophysiological and psychological addiction to drugs of abuse [75–77]. Burst firing of DA neurons appears to depend critically on afferent input mediating inhibition of a slow outward potassium conductance [76, 78–80]. Burst firing of DA VTA cells results in release of the high levels of DA within target accumbal regions required for determination of saliency of a stimulation and to signal prediction of reward. This firing pattern with associated high levels of DA release is critical for development of goal-directed behaviors such as addiction [21, 81, 82]. The importance of this pattern of firing in shaping behavior was confirmed recently in the intact brain using optogenetic tools to drive the firing rate of DA VTA neurons while monitoring successful behavioral conditioning using a learning assay commonly used in behavioral addiction research [83]. Animals can be conditioned to prefer to be in a space where they previously have experienced drugs of abuse. This behavioral paradigm is called conditioned place preference (CPP) and serves as an indicator of the reward experienced by the conditioning drug tested. When burst firing is triggered by light pulses directed to VTA cells transfected with the cation channel channelrhodopsin-2, optogenetically modified mice display CPP by developing a preference to be in the chamber where this light pulse-induced firing pattern was elicited, even in absence of any drug-related conditioning stimuli [83]. These data strongly support a role for DA VTA burst firing in development of addiction-related behaviors. The role played by the LDT in DA VTA cell burst firing was elucidated by attempts to clarify a disparity between *in vivo* and *in vitro* electrophysiological data. While burst firing could be elicited *in vivo* by stimulation of glutamate-containing VTA afferents or application of glutamate agonists and blocked by glutamate receptor antagonists [75, 84–86], this type of firing pattern could not be elicited in DA VTA neurons in brain slices. *In vitro*, DA VTA neurons exhibit a more tonic rhythmic firing which cannot be shifted to a phasic pattern even upon application of glutamate agonists [78]. These data indicated that phasic firing of DA VTA cells relies on synaptic connectivity severed within the reduced preparation of a brain slice. Use of *in vivo* animal models, coupled with excitotoxic or pharmacologic lesion of pontine regions, served to identify a likely necessary controller of behaviorally relevant VTA firing. In an elegant series of studies, it was established that the LDT serves as a “gate” of DA VTA firing. A functioning LDT allows excitatory glutamate input, sourcing perhaps from the PPT, to switch the firing pattern of DA VTA neurons from a rhythmic pattern to a burst firing pattern, as suggested by the finding that local application of glutamate was unable to shift the firing pattern of DA VTA cells following silencing of input from the LDT [87]. Although not definitively established, it is likely that it is cholinergic input from the LDT that serves as the responsible neurotransmitter gatekeeper. This conclusion is based primarily on findings that burst firing of DA VTA neurons is mostly absent in neuronal recordings from a subtype selective nicotinic ACh receptor (nAChR) knockout mouse [88]. The *β*2 nicotinic receptor located putatively on DA VTA cells was shown to be critical to excitability of DA VTA neurons as the nicotine-induced switch from tonic firing to burst firing was prevented in mice lacking the *β*2 receptor but rescued in animals in which the *β*2 receptor was restored selectively to the VTA via local injection of lentiviral vector [89]. Interestingly, the restoration of the *β*2 subunit to the VTA did not fully recover the wild-type phenotype, in that sustained firing rates of DA VTA neurons seen in control conditions were not replicated and a significant delay was noted in acquisition of self-administration of nicotine when compared to time to acquisition in wild-type mice. The authors believe that these alterations indicated the role played by the *β*2 nAChR subtype in other excitatory structures, which they suggest may encompass the afferents from the LDT [89]. 

Taken together, data suggest that cholinergic input directed to the VTA mediates behaviorally relevant excitation of DA VTA cells. However, cholinergic input is also directed to non-DA VTA neurons. As muscarinic ACh receptor activation of GABAergic VTA neurons results in an enhancement of GABA release [90, 91] and nAChRs are present on GABAergic VTA neurons, it is possible that cholinergic stimulation of the VTA could lead to the inhibition of burst firing of DA neurons by activation of GABA-containing cells and subsequent GABAergic inhibition of postsynaptic DA VTA cells. Endogenous cholinergic input to GABAergic VTA cells has been suggested to serve to dampen baseline DA activity during periods with nonarousing stimuli and low levels of ACh release [92, 93]. However, nAChRs exhibit differing activation and desensitization kinetics depending on composition of the subunits comprising the receptors [94, 95], and different subunit compositions of nAChRs on VTA neuronal populations may provide a condition-specific disinhibition of DA VTA cells via differential desensitization when receptors are exposed to high levels of cholinomimetics. Specifically, rapid desensitization of the nicotinic receptors located on VTA GABAergic neurons was believed to play a role in modulation of DA neuronal firing upon nicotine exposure. Rapid desensitization of the nAChRs on GABAergic VTA cells explained why exogenous cholinergic input to the VTA favors DA cell excitation despite activation of nAChRs on cells inhibitory to DA neuronal excitability [92, 93]. Studies using acetylcholinesterase inhibitors suggested that endogenous ongoing cholinergic transmission, likely from the LDT, contributes to the baseline excitation of GABAergic neurons and that exogenous application of nicotine via cigarette smoking leads to a rapid desensitization of the nAChRs located on GABAergic VTA cells. Rapid desensitization by nicotine would make these receptors unavailable to subsequent endogenous cholinergic excitation from the LDT, thereby leading to a drug-induced disinhibition of local DA-containing VTA cells, allowing them to exhibit long-lived excitation [92]. However, evidence for necessity of a more active role of the GABA VTA cells in the nicotine-induced burst firing pattern of DA VTA cells, possibly driven in part by ongoing or drug-stimulated cholinergic input from the LDT, has recently been provided with use of lentiviral expression systems [96]. In that study, putative GABAergic cells did not exhibit reductions in nicotine-induced firing suggestive of nAChR desensitization upon repeat nicotine exposure. Further, only presence of GABAergic cell firing, conducted in a delicate balance with DA VTA neuronal firing led to DA VTA burst firing necessary for nicotine reinforcement [96]. Endogenous cholinergic input played a role in GABAergic firing and therefore was paramount in control of burst firing of DA-containing cells within the VTA [96]. Therefore, instead of interpreting the cholinergic activation of GABA VTA neurons as countering DA VTA cell burst firing, activation of these cells may instead be necessary to drive behaviorally relevant DA VTA cell firing necessary for determination of stimulus saliency. Findings from various studies examining GABA VTA cell firing induced by nicotine exposure remain to be reconciled. In addition, the precise firing activity of VTA GABAergic neurons that is involved in development of addiction-related behaviors beyond those activated by nicotine and the role these cells play in maintenance of chronic responses to drugs of abuse also remain to be further clarified. However, data from various studies lead to the conclusion that ACh input directed to DA and non-DA VTA cells would elicit direct and indirect cellular actions. Further, a role of cholinergic activity within the VTA directed to DA and non-DA cells, presumably sourced from the LDT, is strongly implicated by several studies as involved in behaviorally relevant firing patterns. 

## 6. Behaviorally Relevant Firing Patterns of the LDT

At this time, there have not been any reports of studies using animal laboratory behavioral models used to examine processes underlying the different stages of the addiction cycle concurrent with electrophysiological monitoring of the firing behavior of LDT neurons *in vivo*. Certainly, it would be interesting to monitor the firing of these cells upon first time exposure to drugs of abuse, chronic administration of addictive drugs or reinstatement of drug use following a period of abstinence to determine how the behavior of these cells alters in association with these drug exposure experiences. However, while such studies have not been reported, many studies have been conducted examining LDT neuronal firing across behavioral state in order to elucidate the role of these neurons in controlling states of arousal, wakefulness, and REM sleep. Activation of rostral projections of the cholinergic LDT neurons to the thalamus, the hypothalamus, and the basal forebrain leads to cortical activation during wakefulness and REM sleep [25, 27, 29, 97]. LDT neurons exhibit differential firing patterns that can be associated with the level of arousal of the organism. Recordings from the LDT revealed a population of putatively cholinergic cells that fired in association with cortical activation during naturally cycling periods of wakefulness, non-REM sleep, and REM sleep [38–40, 60]. A subpopulation of these cells with periods of firing preferential to REM sleep was detected [38–40, 60]. The high activity of these cells during REM sleep supports findings of highest cFos levels in cholinergic neurons during this behavioral state [98]. Extracellular recordings of identified cholinergic, GABAergic, and putative glutamatergic LDT neurons were conducted in urethane-anesthetized rats before and during activation of the EEG by somatic stimulation. While all LDT cholinergic neurons switched from low firing to moderate firing levels with EEG activation, roughly half of the GABAergic cells and putative glutamate-containing neurons increased their discharge, with half of each cell type decreasing ongoing firing [99]. All cholinergic neurons recorded in nonanesthetized head restrained mice exhibited an increase in firing rate upon the transition from NREM sleep to REM sleep [100]. While many questions remain regarding the role each LDT cell phenotype plays in state control and naturally occurring levels of arousal, it appears clear that periods of EEG activation are driven by high firing of cholinergic cells, perhaps regardless of afferent targets although this was not a point experimentally addressed [100]. These findings suggest that drug use and drug seeking, both highly aroused states, would be associated with high firing of cholinergic LDT neurons. However, this point has not been directly examined. While it is not known whether cholinergic neurons which project to the thalamus and are involved in EEG activation also send a predominant projection to the VTA, a subpopulation of cholinergic cells in rat was found to send projections to both targets [58]. In addition, the authors noted that while detection of dual projections was relatively infrequent, perhaps the numbers were underestimated in their study, and the possibility of existence of variation across species was presented [58]. If the cholinergic neurons, which send dual projections inclusive of the VTA, are involved in mechanisms of arousal via targets in the thalamus, high firing induced by arousing stimuli may also result in ACh release from VTA collaterals. Alternatively, data collected in a small population of cells from the caudal, mid, and rostral LDT (10 total from the LDT and the sub-LDT [99]) suggest that arousing stimuli may result in activation of the majority of cholinergic neurons, regardless of location within the nucleus or projections outside the nucleus. These findings suggest that arousal induced by drug stimuli may result in activation of virtually all cholinergic LDT neurons, including those which participate in the LDT-VTA pathway. Cholinergic neurons could also become activated via indirect actions as arousal-related activity within the LDT may alter activity in neighboring or contralateral cellular populations via extensive local and bilateral innervation [101]. 

In addition to stimulation of LDT neurons via induction of arousal, drugs of abuse are likely to activate LDT neurons directly (see later section). Enhanced cholinergic activity within the LDT induced by direct actions of abused drugs, coupled with arousal associated with drug seeking and use, and mediated by LDT activity would suggest a high degree of cholinergic output directed to the VTA upon exposure to addictive drugs. The drug-stimulated increased release of ACh within the VTA may lead to heightened levels of DA efflux from the VTA. The synergy of these actions would likely enhance the neurobiological experience of reward induced by the drug exposure, as higher DA efflux would be expected to lead to elevated experience of euphoria. Further studies are needed in which the firing of cholinergic and noncholinergic LDT neurons across the various stages of the cycle of drug addiction are monitored in combination with determination of ACh and glutamate release within the VTA. Of particular interest would be determination, using established behavioral criteria, of whether animals that exhibit different propensities to addict to drugs of abuse show differential firing of subpopulations of cells of the LDT upon drug presentation, use, and withdrawal. Relevant to this issue, in preliminary studies, we have found that the nicotine-induced excitability of cholinergic LDT neurons is enhanced in juvenile mice versus young adult mice. The enhanced excitatory cellular response to nicotine in young animals could be expected to result in greater cholinergic outflow to the VTA contributing to an increased excitation of DA-VTA cells. If cholinergic activation of DA-VTA neurons is importantly involved in development of drug dependency, these cellular findings correlate well with the increased vulnerability exhibited by adolescents to addict to nicotine and, possibly, other drugs of abuse [102–104]. 

## 7. The Behavioral Data That the LDT Plays a Role in Addiction

Anatomical connectivity and functional physiology studies suggest that excitatory input from the LDT is directed to the VTA and that LDT activity results in a release of heightened levels of DA from the VTA to nAcc targets that are associated with assigning saliency to stimuli, and therefore suggestive of being behaviorally relevant. Behavioral studies have collaborated the conclusion that the LDT plays a role in the neurobiology underlying addiction to drugs of abuse. Exposure to amphetamine induces behavioral phases of stereotypy consisting of repetitive movements and locomotor hyperactivity. Repeated doses of amphetamine produce behavioral sensitization of the phase of stereotypy as well as poststereotypy locomotor hyperactivity. As behavioral sensitization is believed to underlie some of the aspects of drug addiction, experimental paradigms monitoring these motoric signs are used in drug addiction studies to indicate a drug's positive reinforcing effect. Attenuation of poststereotypy locomotor hyperactivity was noted in LDT-lesioned, repeat amphetamine treated animals, suggesting a role of the LDT in development of this drug-induced behavior [72]. In another study, lesion of the LDT was found to reduce sensitization of stereotypy in repeat amphetamine-treated rats but did not have an effect on locomotion [105]. In contrast, higher intensity stereotypy was noted in LDT-lesioned repeatedly amphetamine-treated animals [65]. The heightened drug-induced motoric behavior was associated with high levels of accumbal DA [65]. Differences between the duration of monitoring of the animals in the two studies were noted as an explanation for the discrepancy between the findings of the two studies [105]. While the LDT is clearly implicated in the sensitizing effects of psychostimulants, further clarification of the role played is necessary. Stereotypy and locomotor effects are also induced by other drugs of abuse, such as opiates and that nicotine, and these actions can similarly become sensitized as revealed upon repeated exposures. Decreased stereotypy accompanied by a decrease in accumbal DA efflux was found in LDT-lesioned animals when exposed to morphine [65]. Sensitization of the locomotor response induced by repeat nicotine exposure was lower in LDT-lesioned rats [106]. Blockade of AMPA/kainite glutamate receptors in the LDT was found to reduce the reinstatement of drug-seeking behavior following a repeat exposure to cocaine [107]. However, the exact role of the LDT in this behavior is difficult to determine, as probe placement was likely to have included a nearby nucleus, the pedunculopontine tegmentum. Taken together, the behavioral experiments suggest that the LDT mediates several psychobiological actions of several drugs of abuse known to act through the VTA and that the LDT is involved in the induction process of sensitization of behaviors indicative of repeated exposure to drugs with addictive properties. In fact, it has been suggested that the processes underlying the initiation of sensitization might occur at synapses formed by the LDT and DA-containing VTA cells in the mesoaccumbal pathway although this point has not been explicitly addressed [108]. 

Further examinations of the role of firing of cells of the LDT in drug addiction-associated behaviors are currently being conducted with the use of optogenetics which provides an elegant method to activate discrete populations of neurons which share a projection target. Recently, a study similar to that performed within the VTA [83] was presented in which optogenetic activation of LDT neurons was used to test whether stimulation of LDT cells which send afferents to the VTA could lead to acquisition of CPP in lieu of the traditional conditioning stimulation or reinforcer utilized with this paradigm: an addictive drug [73]. The channelrhodopsin-2 cation channel was conjugated with a retrovirus and injected into the VTA. [73]. Presumably, within the LDT, only those LDT cells that contributed afferents to the VTA-LDT pathway were infected. Light pulses were directed to the LDT and capable of activating only those LDT neurons infected with the channelrhodopsin-2 cation channel. Flashes of light directed to the LDT-induced CPP [73]. The pattern of light flashes was found to be important. While phasic pulses of light were successful in inducing CPP, low-frequency pulses failed to elicit a similar effect on conditioning behavior. While other evidence from this study suggested that glutamate projections may underlie this phenomenon, the experimental design was not such that it was possible to selectively activate one phenotypic projection pathway from the LDT to the VTA and monitor presence of CPP. Nor within this study was it possible to test whether acquisition of CPP behavior could be blocked with either local application of glutamate or acetylcholine receptor antagonists. Therefore, identification of the neurotransmitter content of the LDT projection critical to development of CPP was not determined. In addition, while this study is highly suggestive that a glutamate projection was responsible for induction of behavior, even if the responsible projection had been so identified, it must be considered that CPP induced by drugs of abuse could utilize different LDT cellular mechanisms than those activated in this study by light flashes. Interestingly, light pulse stimulation of the LDT of optogenetically manipulated mice did not enhance open-field exploration, a model of anxiety, or increase locomotor activity [73]. If glutamatergic pathways were stimulated, this finding could refute a previous suggestion that the glutamatergic projection from the LDT contributes to amphetamine-induced increases in locomotion [109]. However, a role for the glutamatergic pathway was suggested in default because despite findings that rises in ACh were elicited in the VTA by amphetamine, a role for the cholinergic LDT pathway in drug-induced increases in locomotion and in DA efflux within the nAcc was ruled out [109]. However, the possibility of masking of the participation of cholinergic mechanisms was discussed. The ability of amphetamine to induce rises in DA independent of neuronal electrical stimulation derives in part from actions of this stimulant on reversal of the dopamine transporter so that high levels of dopamine are released from terminals independent of membrane voltage changes. Although amphetamine induced rises in ACh within the VTA from LDT afferents, cholinergic enhancement of DA release may have been present, but undetectable against the high levels induced by reversal of the transporter [109]. Therefore, it was concluded that LDT sourced ACh within the VTA may still be involved in amphetamine-associated behaviors. At this time, the role of ACh in drug-associated increases in locomotor behaviors remains an open question. 

Remaining unanswered questions indicate that studies need to be conducted to further elucidate the role of the LDT in development of drug addiction-associated behaviors. Such studies should utilize drugs of abuse with distinctly different mechanisms of actions as well as utilize several different addiction models to determine if commonalities in activation of LDT populations occur and whether differences emerge with different behavioral models. In addition, recordings of firing behavior of LDT neurons during development of addiction-like behaviors with various drugs could be obtained. The firing patterns could be used in a playback fashion as the conditioning stimulus using electrodes or light pulse stimulation in optogenetically modified mice in drug-naïve conditions to determine whether such neuronal firing behavior induces CPP, or drug exposed associated behaviors using other behavioral models, in absence of drug. Taken together, these studies could evaluate the role of the LDT in induction of behavior induced by drug stimuli. Another possible test of the role played by the LDT in establishment of drug addiction could be conducted by examination of whether addiction behaviors can be elicited by a drug not considered addictive if paired to particular firing patterns of subpopulations of neurons of the LDT, such as those that project to the VTA. In addition, at a particular concentration, not all individuals addict to drugs of abuse following exposure. The route of exposure can also determine the ability of a drug to become addictive. A similar phenomenon exists in animal models and across species and it could be determined whether stimulation of the LDT can induce drug preference in an animal previously determined, either based on amounts of drug exposure or route of administration, to be resistant to drug addiction-associated behavioral conditioning. At this time, it is unclear what contribution separate populations of LDT neurons have on development and maintenance of addictive behaviors. Some studies provide strong evidence that the glutamatergic LDT neurons are involved in development of behavior, whereas others convincingly implicate the ACh-containing neuronal population, especially in controlling behaviorally relevant DA VTA firing. Therefore, future experiments should be conducted, perhaps with use of optogenetic methodology and phenotypic selective transfection to drive firing selectively of cholinergic or glutamatergic LDT neurons concurrent with recordings within the VTA and behavioral monitoring. In this way, it might be possible to elucidate the involvement of each cellular phenotype in addiction-associated behaviors and explore whether distinct roles are served by separate populations of LDT neurons. It is fully expected that with the tools currently available to addiction researchers, the role of different populations of cells within various neuronal groups in development of the various addiction-associated behaviors will be more fully elucidated. 

## 8. Direct Cellular Actions of Drugs of Abuse on LDT Neurons

If the LDT is involved in behavioral actions induced by drugs of abuse via activation of VTA DA cells, it is necessary to understand how drugs of abuse influence LDT cellular functioning. If endogenous excitatory cholinergic mechanisms within the VTA are involved in production of firing activity of DA VTA cells required for development of neurobiological addiction, the most parsimonious hypothesis is that drugs of abuse result in activation of the population of LDT cholinergic neurons projecting to the VTA, leading to heightened release of ACh to this midbrain target. In addition, drugs of abuse could activate glutamate LDT cells contributing to the LDT-VTA pathway. Few studies have been conducted examining the cellular actions of drugs of abuse on LDT neurons and fewer still have focused on whether exposure to drugs of abuse induces locally mediated changes in LDT neuronal activity. One of the earliest studies of the actions of drugs of abuse on cells of the LDT examined the ability of a single subcutaneous injection of nicotine to induce expression of cFos within cells of the LDT [110]. Acute exposure to nicotine did result in higher levels of expression of cFos within neurons of the LDT. However, counterstaining with NADPH, a marker of cholinergic cells, revealed that the majority of cFos positive cells was noncholinergic. These findings suggested to the authors that nicotine exerted direct actions within the LDT at nAChRs located on noncholinergic neurons, presumably glutamate- or GABA-containing cells. However, it was not ruled out that nicotine stimulated activation of these noncholinergic cells could indirectly influence activity of neighboring cholinergic neurons depending on whether directly activated cells exhibited projection profiles onto these local neurons. 

However, arguing for a direct effect of nicotine on cholinergic LDT neurons, a nAChR subunit study showed coexpression of mRNA for the *α*7 and *β*2 subtypes within cholinergic LDT cells. While presence of mRNA does not necessarily mean translation into functional protein and proper insertion of nAChRs into the membrane, these data did suggest that nAChRs could be present on cholinergic neurons and nicotine could exert direct actions on ACh-containing cells. Direct actions could act in concert with indirect actions if, in addition to a postsynaptic localization, nAChRs were present on terminals directed to the postsynaptic cell [111]. Supporting this conclusion and demonstrating presence of functional nAChRs on cholinergic LDT neurons, patch clamp recordings from mouse brain slices combined with immunohistochemistry showed that nicotine has direct excitatory actions on the membrane potential of cholinergic cells, inducing a large inward current sufficient to induce action potential firing [112]. In addition, while GABAergic inhibitory postsynaptic currents (IPSCs) directed to cholinergic cells were stimulated by nicotine, a larger proportional increase of glutamatergic excitatory postsynaptic currents (EPSCs) was induced by nicotine, presumably via actions of nicotine on nAChRs located on presynaptic glutamate-containing terminals directed onto cholinergic cells. The differential presynaptic actions of nicotine would serve in combination with direct effects to heighten the excitatory action of this drug on cholinergic LDT cells [112]. Use of nAChR subunit-specific antagonists suggested that nAChRs located on cholinergic soma mediating nicotine-induced inward currents were comprised of *α*7, *β*2, and non-*α*7 subunits, agreeing with the mRNA expression study. The exact subunit composition of nAChRs located on GABAergic and glutamatergic LDT neurons is not known; however, mRNA for the *α*4 nAChR was detected in noncholinergic neurons, suggesting inclusion of this subunit in at least some of those receptors present on these cells [111]. Electrophysiological studies in combination with application of pharmacological receptor antagonists revealed an interesting difference between nAChR subunits mediating the IPSCs and EPSCs directed to cholinergic LDT neurons. Glutamatergic EPSCs directed to cholinergic cells were sensitive to MEC, a non-*α*7 nAChR antagonist, with insensitivity to both MLA, an *α*7 subunit receptor antagonist, and DH*β*E, an antagonist with actions at *β*2 subunit-containing nAChRs. These findings suggested that glutamatergic terminals impinging on cholinergic neurons, the source of which is unknown, contained nAChRs lacking both *α*7 and *β*2 subunits. In contrast, GABAergic-mediated IPSCS were sensitive to all three drugs, indicating the presence of homomeric *α*7 subunit-containing nAChRs and heteromeric nAChRs-containing *β*2 and non *α*7 subunits. While the subtypes of nAChRs on GABA- and glutamate-containing neurons impinging on DA VTA neurons were also found to differ across these two cell phenotypes [92], the compositions of nAChRs on GABA- and glutamate-containing terminals impinging on cholinergic LDT neurons was different from those of nAChRs present on excitatory and inhibitory terminals impinging onto DA VTA cells. Within the VTA, DA neurons receive input from glutamatergic terminals with nAChRs comprised of *α*7 subunits [92]. This subunit was absent in nAChRs located on GABAergic presynaptic inputs [92]. The difference in desensitization kinetics between nAChR receptors lacking or containing the *α*7 subunit could explain how nicotine can result in prolonged activation of DA VTA neurons despite the rapid desensitization kinetics of nAChRs located on DA VTA cells. High affinity nAChRs on GABAergic inputs would rapidly activate and rapidly desensitize in the presence of nicotine concentrations typically experienced by a smoker whereas, the nAChRs on glutamate cells would exhibit kinetics typical of *α*7 subunit-containing receptors, with a reduced activation and slower desensitization kinetics [92]. The resultant nicotine stimulated changes in balance of inhibitory and excitatory presynaptic input, with a quick drop out of nAChR stimulated GABAergic cell activity, concurrent with a longer-lived excitation of nAChRs on glutamate-containing cells would summate to enhance excitatory glutamate release leading to a long-lived excitation of DA VTA cells, the duration of which coincides with the spacing of nicotine intake exhibited by a smoker [92]. Although this scenario was quite compellingly supported by the data, a more active role of GABAergic VTA cells in behaviorally relevant activity patterns of DA VTA cells has been recently suggested [96]. Although a similar mechanism of heightening glutamate transmission does not seem to be operating in the LDT, different subunit composition of nAChRs on cholinergic and across noncholinergic terminals directed to these cells could result in very different physiological and behavioral actions upon exposure to nicotine or the endogenous agonist, ACh. In addition, direct recordings of noncholinergic LDT cells and precise determination of the identity and stoichiometry of nAChR subunits comprising receptors located on cell somas could also reveal a basis for differential activation by nicotine due to distinct functional properties such as high or low sensitivity to activation [113] or differential agonist desensitization, which may contribute to behavioral effects and reinforcement of its use. Although precise mechanisms of action of nicotine within the LDT remain to be elucidated, clearly nicotine evokes strong excitation locally within the LDT neuronal population. 

Direct cellular actions on cholinergic LDT neurons of another drug which exhibits an abuse potential have been recently examined. GHB (*γ*-hydroxybutyric acid) is a natural chemical that works as a neurotransmitter within the human brain. Exogenous application of GHB is currently used therapeutically as a sedative taken before bedtime in individuals with narcolepsy. It is believed that one way by which the drug exerts its clinical actions is via consolidation of sleep in this population of individuals who experience highly fragmented nighttime sleep [114]. However, its neuroactive effects must stem from a more mechanistically complicated basis because while sleep is consolidated the first night of exposure, clinical effectiveness of this drug in reducing excessive daytime sleepiness is achieved following several weeks of treatment. Interestingly, GBH has emerged as a drug of abuse, with a mild addictive potential heightened in individuals with a history of alcohol abuse [115, 116]. Although some evidence supports existence of a GHB specific receptor, the overwhelming preponderance of data suggests that GHB exerts its physiological and psychological actions via activity at the GABA_B_ receptor [117–120]. Studies of actions of GABA on cells within the LDT revealed that cholinergic LDT neurons contain GABA_B_ receptors which when activated by traditional GABA_B_ receptor agonists mediate a long-lived membrane hyperpolarization [121]. As the majority of behavioral and cellular actions of GHB has been found to be mediated by activity at the GABA_B_ receptor, actions of GHB at that receptor within the LDT were examined. Interestingly, GHB had direct inhibitory actions on fewer cholinergic neurons than actions induced by more traditional GABA_B_ receptor-acting drugs. In addition, smaller amplitude inhibitory currents were induced in cholinergic LDT neurons as compared to those induced within cells of a nearby pontine nuclei comprised of serotonergic neurons, the dorsal raphe [122], despite the fact that baclofen, a GABA_B_ receptor agonist elicited similar sized currents in cells from both nuclei. These data indicated that within the LDT, inhibitory effects of GHB would be smaller than predicted based on membrane actions of other GABA_B_ receptor agonists, possibly allowing for more cholinergic activity in the face of exposure to this sedative than would be expected. The finding of a differential action of GHB across different neuronal phenotypes is similar to that found within the VTA, where GHB was found to preferentially inhibit the GABAergic VTA neurons over DA VTA cells via a GABA_B_ receptor mediated mechanism. The preferential inhibition of inhibitory GABAergic VTA neurons may underlie, in part, the cellular mechanism whereby GHB exhibits an addiction potential that is not shared by traditional GABA_B_ receptor agonists such as baclofen [123]. Although speculative, it is possible that lower than expected inhibitory actions of GHB within the LDT may allow cholinergic outflow, to the VTA, which may contribute to conferring a positive saliency and experience of euphoria upon its use. Alcohol, another addictive drug with sedative properties, also induces inhibitory cellular effects via activation of GABA receptors. Alcohol directly stimulates synaptically located GABA_A_ receptors, with a potential contribution of effects on behavior of actions at delta subunit containing GABA_A_ receptors likely to be extrasynaptically located, although this point remains controversial (for review, see [124]). As data suggests that GABA_A_ receptors are present both within synapses and extrasynaptically on LDT neurons [121], it would be interesting to determine whether this addictive drug mediates actions on LDT neurons at these receptors and whether cholinergic and noncholinergic cells are differentially affected. Also of interest would be elucidation of whether alcohol-induced cellular actions differ from those elicited in other brain areas where cellular effects of this drug have been studied. Acute alcohol exposure has been shown to have profound actions on stimulation of the I_H_ current in DA neurons of the VTA with chronic exposure inducing a downregulation of this current [125]. I_H_ current contributes to excitability of a cell and is present on cholinergic LDT cells [126]. If similar actions are induced on I_H_ current on neurons of the LDT by acute and chronic alcohol exposure, these drug-induced changes would be expected to significantly influence cellular excitability and firing patterns of these cells. Although actions of alcohol on GABA receptors within the LDT have not been explicitly reported, in a very interesting preliminary study, alcohol was reported to attenuate membrane inward currents mediated by *α*7-subunit nAChRs [127]. In contrast, nAChR currents mediated by non-*α*7-containing nAChRs were potentiated [127]. These data suggest a dual action of alcohol on nAChR receptors within the LDT depending on the constellation of subunits present and beg further explorations to clarify the cellular actions of alcohol on LDT cells. In summary, at this time, there are few studies examining the direct cellular actions of drugs of abuse across the population of LDT neurons. However, as drug use certainly exposes the entire brain to the actions of that drug, the few studies that have been conducted strongly suggest, in combination with indirect data provided by findings that ACh levels rise in the VTA upon exposure to drugs of abuse like amphetamine [109], that addictive drugs would have actions on cholinergic and noncholinergic LDT neurons sufficient to alter outflow from this nucleus. Further, it is quite possible that these cellular actions could play a substantial role in the assignment of saliency of stimuli via activation of neurons contributing afferents to the LDT-VTA pathway. A very interesting question that arises from the few studies that have been conducted is whether drugs of abuse which exhibit relatively low addictive potential activate neurons of the LDT to a lesser degree than drugs associated with high addictive liability. 

## 9. Intrinsic Membrane Properties Can Influence LDT Neuronal Firing

Two factors shaping the electrophysiological status of neurons are the afferent input directed to the cells and the intrinsic membrane properties of those cells. Firing of LDT neurons involved in drug addiction physiology is almost certainly driven by a combination of afferent input activated by drugs of abuse and by the actions of drugs of abuse on the cell's intrinsic membrane properties. Early experiments conducted with intracellular recordings in guinea pigs revealed that cholinergic LDT neurons presented with different electroresponsive characteristics. Most were capable of firing either tonically or in bursts [128]. Presence of a prolonged afterhyperpolarizing potential mediated by a potassium conductance or a low threshold calcium conductance was believed to underlie different observed firing modes of subpopulations of these cells, such that one conductance limited the maximum firing frequency achieved by the cells and the other contributed to cellular burst firing. Subsequent whole-cell patch clamp studies confirmed that when considering intrinsic firing properties, the LDT is not a homogenous nucleus. Neurons of the LDT can be broadly classed into three types, based on their intrinsic electrophysiological properties as examined in rat and guinea pig [129–132]. Type I neurons display a low threshold calcium spike, I_T_, which allows a burst firing pattern. Type II cells lack the low threshold spike present in Type I cells and display an outward potassium current, which has been designated I_A_. When this potassium current is activated, it serves to hyperpolarize the cell, resulting in cellular inhibition and a dampening of excitatory input. Both types of currents are present in Type III cells. While the current profiles of Type I and Type III cells indicate they would be highly active, inactivation kinetics of channels mediating ionic conductances can play a large role in the participation of the current underlying low threshold spiking with a potential contribution of modulation of currents by intracellular calcium levels. Therefore, prior membrane voltage activity can promote a brake on spiking until recovery of the current is achieved. These factors indicate that activity of these cells would be determined by prior membrane experience, making relevant predictions of outcomes of stimulation of these currents difficult [133–135]. A large proportion of the Type II and Type III LDT cells have been identified as being cholinergic, with Type I cells likely to be noncholinergic [131, 136]. The actions of drugs of abuse on the activation or inhibition of the conductances used to classify neurons within the LDT have never been examined, but almost certainly effects on these currents are elicited as drugs of abuse have been found to affect various conductances when this point has been studied in other cell types. Intoxicating levels of alcohol have been shown to differentially affect subpopulations of striatal neurons depending on the ionic conductance(s) present within each cell type. Low threshold burst neurons were inhibited by alcohol via activation of a calcium activated potassium conductance. Similar concentrations of alcohol induced a depolarization via inhibition of an inwardly rectifying potassium conductance in high frequency bursting cells within the striatum; whereas, alcohol had no action on membrane conductances within a third type of striatal neuron [137]. In preliminary studies, we have found that the I_H_ current, which exhibits a developmental expression within the LDT paralleling that seen in cells of the PPT [138], is decreased in cholinergic neurons by acute exposure to nicotine. Almost certainly differential actions of various drugs of abuse on cellular conductances are likely to exist across the heterogeneous LDT neuronal population. In addition, the proportion of cell types of LDT neurons varies with age [126] and may also underlie in part different responsiveness of the entire neuronal network to drugs of abuse across different developmental stages. Further examination of how drugs of abuse influence membrane conductances present across the membrane of LDT cells needs to be conducted to determine whether it would be expected that drugs of abuse would alter the activity of channels underlying these conductances, which would significantly alter the excitability of these cells. In addition, it needs to be determined whether further distinction of cell type by electrophysiological signatures beyond those established can be conducted and whether a particular cell type correlates with those that send projections to the VTA. If this was found to be the case, the possibility exists that the typing of a cell based on its intrinsic membrane properties could be used to classify whether an LDT neuron is likely to be involved in drug addiction processes via differential afferent targets. 

## 10. Actions of Neuroactive Substances on LDT Neurons

While drugs of abuse can influence LDT cellular activity directly, they are also likely to influence LDT activity indirectly via actions on afferent inputs directed to LDT cells. Such activity could result in inhibition, or promotion, of release of neuroactive substances onto postsynaptic LDT neurons. Certainly nicotine acting at nAChRs located on presynaptic GABAergic and glutamatergic terminals results in alterations of glutamate and GABA release upon cholinergic and noncholinergic LDT cells. This effect is so pronounced that it is likely to strongly shape the output to target neurons [112]. LDT neurons receive contact from serotonergic, histaminergic, cholinergic, noradrenergic, adenosinergic, orexinergic and other peptide-containing terminals [41, 45, 46, 139, 140]. These terminals arise both from projection neurons deriving from other nuclei and from neurons located within the LDT [41, 45, 46, 139, 140]. In addition to stimulating receptors on local terminals, drugs of abuse have been shown to activate several of the neuronal regions which provide input to the LDT. Stimulation of the widespread neuronal regions that send input to the LDT sufficient to result in release of neuroactive substances within synapses in the LDT could certainly influence LDT firing. For example, nicotine applied locally to dorsal raphe neurons, which provide the majority of neuronal serotonergic projections, was found to result in decreases in firing rate of LDT cells, presumably via inhibitory actions of serotonin released from afferents directed to the LDT [141]. Accordingly, it is important to consider what the resultant action on LDT cells would be of stimulation of afferents by use of addictive drugs. Focus of cellular actions of neuroactive compounds within the LDT has centered on membrane actions of these agents on cholinergic neurons. Neurons in these studies were identified as being acetylcholine-containing by presence of NADPH or neuronal nitric oxide, which has been shown to colocalize with ACh in 99% of LDT cells [142]. *In vitro* patch clamp studies revealed that serotonin inhibits cholinergic LDT neurons by stimulation of an inwardly rectifying potassium conductance most likely secondary to activation of the 5-HT_1A_ subtype serotonin receptor [130, 131]. Noradrenaline (NA) also inhibits cholinergic LDT cells via activation of an inwardly rectifying potassium conductance secondary to stimulation of the alpha-adrenergic receptor [143]. NA-mediated inhibition may mask a cooccurring excitation of these cells [143]. While the cellular mechanisms behind this action have not been extensively studied in the LDT, immunohistochemical localization of a differential distribution of adrenergic receptors across cholinergic LDT neurons may be involved. One-half of the identified cholinergic neurons in the LDT were found to be labeled for the *α*_2A_ adrenergic receptor, whereas label for the *α*_1A_ receptor was detected in one third of examined cholinergic neurons [144]. As activity of the *α*_1A_ receptor is associated with cellular excitation in response to NA [145], excitatory responses evoked by NA in cholinergic LDT neurons may be mediated by activation of this receptor type. The differential localization of noradrenergic receptors across cholinergic LDT neurons may underlie different projection patterns of these cells and involvement in divergent behaviors. This finding suggests the possibility that presence or absence of subtypes of noradrenergic receptors could be useful for cholinergic LDT cell categorization. As adenosine is involved in suppressing arousal, the cellular actions of adenosine have also been examined within the LDT. Adenosine inhibits cholinergic LDT neurons via activation of an inwardly rectifying potassium conductance [146]. In addition, adenosine inhibits an I_H_ current, which could be expected to contribute to further inhibition of firing of cholinergic neurons [146]. An intimate association between sleep and the immune system leads to studies examining actions of interleukin-1 on cholinergic LDT cells. Interleukin-1 inhibits the firing rate of LDT neurons, putatively identified as cholinergic based on their firing profiles [147]. Another neurotransmitter that may exert an inhibition of cholinergic neurons was found surprisingly to be glutamate. Although glutamate is the major excitatory neurotransmitter in the brain when acting at ionotropic glutamate receptors, activation of metabotropic glutamate receptors (mGluRs) of the Group I, type 5, and Group 2 (type 2/3) elicited a relatively long-lived membrane hyperpolarization in cholinergic LDT cells [148]. These data suggested a dual polarity action of this classic excitatory neurotransmitter with final actions likely dependent on receptor distribution at the target site. Direct actions of DA on LDT neurons have not been reported, but local application of DA had little effect when firing of extracellular units in the cat LDT that were presumed to be cholinergic was monitored [149]. These data suggest that DA input directed to the LDT, perhaps via activation of DA VTA neurons which project to the LDT [3, 15], might not exert strong direct actions on membrane potential. However, DA might have more subtle cellular effects upon neurons of the LDT as suggested by findings that DA receptor knockout mice exhibit alterations in behaviors that are controlled in part by the LDT (for review, see [150]). 

Several neuroactive compounds have been found to excite cholinergic LDT neurons. Molecular and functional evidence for presence of glutamaceptive AMPA, NMDA, and kainate receptors on cholinergic neurons have been presented [151–153], and not surprisingly, glutamate excites cholinergic neurons via activation of ionotropic glutamate receptors. In brain slices, endogenous release of glutamate onto cholinergic neurons is apparent, indicating a high level of ongoing glutamate tone directed to these cells remaining intact in this reduced preparation [152, 153]. In addition to glutamate-induced excitatory actions mediated by ionotropic receptors, glutamate can excite cholinergic neurons directly via activation of mGluRs of the Group I, type 1 [148, 154]. Based on *in vitro* findings within the PPT and *in vivo* extracellular recordings in cat, histamine is presumed to excite cholinergic LDT neurons via an H_1_ receptor mediated increase in membrane resistance [149, 155]. In support of this conclusion, although not the main focus, all LDT neurons examined in an *in vitro* patch clamp brain slice study and identified as cholinergic based on immunohistochemistry were found to respond to histamine with a long-lived depolarization and an increase in input resistance [156]. Ghrelin, a peptide importantly involved in stimulation of hunger, was found to depolarize putative cholinergic neurons via decrease of a potassium conductance [157]. The excitation of acetylcholine-containing LDT cells induced by ghrelin may be importantly involved in ghrelin-mediated reinforcement of food intake via cholinergic activation of DA VTA cells involving nicotinic receptors [158]. The arousal promoting peptide, orexin, also depolarized cholinergic LDT neurons primarily via stimulation of one of the two known neuronal orexin receptors, Ox1, resulting in activation of a nonspecific cation current [159, 160]. The orexin-mediated depolarization was sufficient to drive firing of these cells and injections of orexin peptide into the pons resulted in enhancement of ACh release [161]. These data support the conclusion that the arousing actions of orexin may depend, in part, on direct activation of cholinergic LDT neurons which are incorporated in the RAS and project to rostral centers controlling EEG activation. The mechanistic interpretation of the interplay of orexin and cholinergic LDT neurons in mediation of arousal is strengthened by the finding that orexin-containing projections targeted LDT cells projecting to the mPFC and 1/3 of the targeted LDT neurons were positive for ChAT [162]. Both the ghrelin and orexin peptide systems have been suggested as potential targets for treatment of abnormal appetitive behaviors, and it would be of interest to determine whether manipulation of their actions within the LDT represents a viable strategy for such treatments. 

In light of the long held belief that ACh was solely inhibitory to cholinergic LDT neurons, perhaps most intriguing is the finding of two direct actions of ACh on cholinergic cells mediated by two different types of muscarinic receptors, and these two direct actions have opposite effects on the membrane voltage. It has long been accepted that application of muscarinic receptor agonists to cholinergic LDT neurons results in an inhibition of these cells [131, 163]. Inhibition is mediated via activation of the M2 and M4 muscarinic receptor subtypes, as shown definitively by failure of cholinergic agents to induce membrane inhibition of cholinergic LDT neurons in selective muscarinic receptor knockout animals [164]. Presence or absence of inhibitory cholinergic feedback on cholinergic LDT neurons likely plays a significant role in shaping VTA firing and VTA-dependent behaviors. Cholinergic activation of the M2 receptor located on LDT-VTA afferents was believed to be responsible for the decrease in DA efflux seen amongst the excitatory components of DA rises following stimulation of the LDT [64–66]. Further, lack of cholinergic inhibitory feedback may underlie the higher self-administration of cocaine seen in M4 receptor knockout animals, as well as the higher DA efflux noted within the nAcc in response to cocaine exposure [165]. Although cholinergic input may also source from outside the LDT, cholinergic LDT neurons themselves are responsible for an ACh-containing input directed to LDT cholinergic cells. Although cholinergic input within the LDT is directed for the most part to noncholinergic LDT neurons, studies of synaptic profiles have suggested that LDT acetylcholine-containing neurons release ACh onto neighboring cholinergic cells within the ipsi- and contralateral LDT [41, 57, 166]. Inhibitory actions of ACh on cholinergic neurons within the LDT were thought to serve as a feedback mechanism to reduce or terminate ongoing cholinergic activation during periods of LDT-mediated cholinergic activation of the EEG [131, 163]. However, complicating this interpretation, data was recently presented indicating that cholinergic input directed to cholinergic LDT neurons would not be exclusively inhibitory. Cholinergic agonists were found to elicit in cholinergic LDT cells, a late developing membrane excitation [164]. This excitation was often accompanied by glutamate-mediated EPSCs [164]. The two excitatory actions of cholinergic agents may not have been noted in earlier studies, as they are difficult to appreciate due to overlap with the earlier onset, large inhibitory M2 and M4 receptor-mediated current. Using pharmaceutical receptor antagonists, activation of pre- and postsynaptic M1 receptors was suggested to underlie the EPSCs and membrane depolarizing current, respectively [164]; however, given the notorious lack of specificity of muscarinic receptor agents, this point needs to be directly addressed using M1 receptor knockout mice. These findings indicate that the final action of ACh input directed to cholinergic LDT neurons would not be a simple, fast-onset, rapidly desensitizing excitation at nicotinic receptors [112], coupled with a M2 and M4 muscarinic receptor-mediated inhibition, as previously believed [131, 163]. Instead, the effect might depend on the levels of ACh in the vicinity of particular populations of muscarinic receptors, leading to a signal to noise filtering scenario where small excitatory events in cholinergic neurons would lead to a negative feedback inhibition of cholinergic cells, whereas larger excitatory events would lead to further excitation that may extend to the entire cholinergic network [164]. Dual actions of ACh on postsynaptic cholinergic LDT neurons, coupled with a heavy presence of excitatory, ACh-containing synapses directed to neighboring noncholinergic cells [166], suggest that ACh actions within the LDT would be complex. 

Actions of neuroactive compounds on noncholinergic LDT neurons have been less well studied and certainly the neurotransmitter content of the noncholinergic cells was not determined in the majority of these examinations. Orexin was found to be excitatory to noncholinergic LDT neurons, and there was not a difference detected in excitatory actions of this peptide between cholinergic and noncholinergic LDT cells [159, 160]. Effects of NA on noncholinergic LDT cells were mixed, with some cells responding with excitation and some with inhibition [143]. Interestingly, a subpopulation of noncholinergic cells, which were small in size and close in opposition to cholinergic neurons, possibly indicative of their being GABA-containing, responded to NA with a two-phased response. The first response was characterized by a rapid depolarization of the membrane, which was followed by a long-lived barrage of EPSCs. The NA-elicited EPSCs were found to be mediated by an indirect glutamatergic mechanism, suggesting excitation at NA receptors of glutamate-containing LDT terminals directed to the noncholinergic, putatively GABAergic neurons [156]. Histamine depolarized noncholinergic LDT neurons; however, the mechanism of depolarization appears to be different from that activated in cholinergic cells as it was accompanied by a decrease in input resistance [156]. Serotonin application also resulted in depolarization of noncholinergic LDT neurons, which was associated with a decrease in input resistance; however, the conductance underlying this action was not identified [156]. Carbachol, a mixed nicotinic and muscarinic cholinergic receptor agonist, was found to induce depolarization in all noncholinergic neurons tested [156] via actions at muscarinic receptors [164]. As nAChRs have been shown to be located on LDT noncholinergic neurons and their activation results in cellular excitation [112], the findings of muscarinic receptor mediated excitation suggest that the cumulative effect of ACh input directed to cholinoceptive receptors located on noncholinergic LDT neurons would be excitatory. 

The final effect of drugs of abuse on LDT transmission to target regions is not likely to be correctly gauged by examination of membrane actions of individual input directed to LDT neurons. Similarly, the final outflow from the cholinergic and noncholinergic LDT neurons to target regions in response to exposure to drugs of abuse is likely a compendium of multiple actions of abused drugs on a plethora of inputs directed to LDT cells, in combination with direct actions of those drugs on LDT neurons. Certainly, VTA-mediated drug elicited behavior(s) results from an integration of neuronal output from a host of drug-activated neuronal phenotypes. Regardless, while it is unlikely to present the complete picture, it is important to know how drugs of abuse influence afferent input directed to the LDT. With such knowledge, based on studies of cellular actions of neuroactive compounds on cholinergic and noncholinergic LDT cells, we can make predictions regarding how drug-stimulated activation of a particular input is likely to influence LDT cell excitability and firing. At this time, this is a necessary step if models of addiction inclusive of the LDT are to be generated and utilized to inform therapeutic strategies treating drug dependency. 

## 11. Drug-Induced Plasticity of Synapses of the LDT

The compulsive nature of drug abuse which can override a conscious desire to not use and the persistent vulnerability to drug use despite long-periods of abstinence suggest that drugs of abuse induce metaplastic alterations in circuits responsible for processes driving drug-seeking behavior. The underlying mechanisms behind these alterations are likely to involve protein expression and changes in synaptic architecture [167]. Risk of life-long relapse after physical withdrawal symptoms have disappeared suggests these neuronal changes persist in absence of acute actions of the drug. Indeed, convincing evidence has been provided that most drugs which display abuse potential alter strength of synapses on VTA DA neurons and/or synapses within the nAcc and these changes persist for extended periods of time [23, 168]. While most studies of drug induced synaptic plasticity have focused on changes within the VTA-nAcc axis, changes in synaptic strength induced by drugs of abuse have been found in other neural areas, including the amygdala and the basal nucleus of the stria terminalis (for review, see [167]). Interestingly, recent findings suggest that actions on alteration of synaptic structure may also be induced by drugs of abuse within the LDT. The first evidence that drugs of abuse may induce synaptic plasticity within the LDT was provided by the finding that amphetamine sensitized rats showed a larger increase in DA in the nAcc following AMPA administration within the LDT [72]. The enhanced LDT response to AMPA was suggestive of activation of postsynaptic plasticity underlying development of long-term potentiation at LDT synapses [72]. These synaptic changes could contribute to the sequelae of mechanisms leading to the progression to compulsive drug use or reestablishment of use of drugs of abuse after abstinence, since within the same study changes in cellular excitability within the LDT were associated with heightened behavioral sensitization to amphetamine [72]. However, detailed characterization of the site of plasticity was not possible in that study, and therefore, the location of the relevant psychostimulant induced change(s) altering cell firing remains an open question. A recent preliminary report suggested that repeated administration of another psychostimulant, cocaine, failed to induce in cholinergic LDT neurons a change in the AMPA/NMDA glutamate receptor ratio which is a measure used to indicate occurrence of postsynaptic plasticity [169]. In contrast, the paired pulse ratio was altered indicating a persistent presynaptic change resulting in a heightened glutamate release, which could result in a heightened response mediated by postsynaptic AMPA receptors. These data, while preliminary, directly demonstrate presence of drug-induced plasticity within synapses of the LDT [169]. As drugs of abuse do not excite or inhibit neurons by one common mechanism, it will be interesting to determine whether drugs of abuse with mechanisms of action differing from those of the stimulants, cocaine and amphetamine, induce plastic changes at synapses within the LDT. 

As the LDT receives a strong innervation of orexin-containing fibers and this peptide is known to be excitatory to cholinergic and noncholinergic LDT neurons [140], it is interesting to note that orexin induced synaptic plasticity within excitatory glutamatergic synapses directed to DA VTA cells. Activation of Ox1 receptors on DA VTA neurons resulted in translocation of NMDA receptors to the cell surface, resulting in a heightened excitatory effect on the membrane potential upon glutamate exposure [170]. The orexin-mediated increase in NMDA receptors within the VTA could be expected to contribute to LTP and burst firing of DA cells induced by drugs of abuse [171]. Supporting this conclusion, application of an Ox1 receptor antagonist after chronic cocaine treatments blocked synaptic DA VTA neuronal plasticity [171]. Drug use and drug-seeking behaviors are associated with a high degree of behavioral arousal, which could be promoted by orexinergic mechanisms as activation of orexin neurons has been associated with drug addiction behaviors [172]. A role of orexin receptors within the VTA in development of such behaviors was shown by the ability of an Ox1 receptor antagonist to block acquisition of behavioral sensitization to cocaine as shown by monitoring of locomotor activity in an open field [171]. Actions of orexin on glutamate transmission have been studied within the LDT. Orexin was found to stimulate glutamate afferents directed to cholinergic LDT neurons via activation of postsynaptic Ox1 receptors [159]. However, the role that orexin input directed to the LDT plays in LDT synaptic plasticity or alterations in NMDA receptor functionality in particular was not specifically examined in this electrophysiological study [159]. There is a basis for such a study, as evidence presented in the VTA would suggest the possibility that similar drug-induced mechanisms could be occurring within synapses of the LDT. As synaptic plasticity is a calcium-dependent process, the finding that activation of LDT Ox receptors induces large increases in intracellular calcium [173] supports the possibility that stimulation of Ox receptors could be involved in synaptic modifications induced by drugs of abuse within the LDT. Further work needs to be conducted to determine whether drugs of abuse induce increases in synaptic strength, either via changes in presynaptic neurotransmitter release machinery or at postsynaptic sites by alterations in receptor numbers, subunit compositions, or functioning. Results from such studies could indicate whether reversal, or blockade, of local plastic changes within the LDT offers a potential treatment strategy directed against the maladaptive learning components of drug abuse behaviors. 

## 12. The Role of Noncholinergic LDT-VTA Pathways in Drug Addiction Neurons

The focus of this review has been on direct and indirect actions of drugs of abuse on cholinergic neurons of the LDT as data from several studies suggest that output from this cell group to the VTA may be important in mediating the addictive properties of drugs of abuse via demonstrated cholinergic regulation of behaviorally relevant firing patterns of DA VTA neurons. However, the majority of cells within this nucleus are not cholinergic, and in fact, the largest proportion of neurons within the LDT is GABA containing, and do not colocalize ACh. A large population of glutamate-containing cells is present which also do not release ACh. Some of these noncholinergic neurons send local projections and make synaptic contact within the LDT and influence neighboring LDT cells of the same, or, of a different phenotype [33, 41]. The role of noncholinergic LDT neurons in control of the excitability of neighboring cholinergic cells has been shown in studies using isolated LDT brain slices in which ongoing glutamate excitation of cholinergic LDT cells could be aborted when the LDT was surgically isolated and local tissue was resected [164]. These findings indicated that the somas of the majority of glutamate-containing cells which provided excitatory input directed onto cholinergic LDT neurons in this preparation were located within the LDT. Drugs of abuse may modulate output of LDT cholinergic cells indirectly via actions on local, noncholinergic cells. For example, nicotine has been shown to activate noncholinergic neurons of the LDT with resultant increases in glutamatergic and GABAergic input directed onto the postsynaptic cholinergic cell, some of which likely derives from local cells [112]. However, some of the noncholinergic cells send projections outside the LDT and may influence behavior via these extra-LDT pathways [33, 41]. Demonstration of symmetrical and asymmetrical synaptic inputs from the LDT to the VTA suggested that the LDT likely sends glutamate-containing and GABAergic projections to the VTA. Interestingly, symmetrical inputs from the LDT to the VTA were shown to be preferentially directed to mesoprefrontal cells and were immunoreactive for GABA, indicating inhibition of the prefrontal pathway by this LDT input [33]. In contrast, asymmetrical contacts from the LDT were directed to mesoaccumbal neurons. This synaptic profile indicated excitatory tone was directed to mesoaccumbal neurons from this pathway, which is likely to influence mesoaccumbal DA neuronal output [33]. While it is believed the LDT axons forming the asymmetrical synapses largely contain ACh [32], certainly they could in part derive from glutamate-containing LDT afferents [33]. Indeed, recent data indicate that glutamate afferents deriving from the LDT provide a major innervation of ventral VTA DA neurons which project to the lateral shell of the nAcc [73]. 

While the neurotransmitter(s) necessary for induction of LDT-involved, light pulse dependent CPP was not determined [73], anatomical findings in the same study demonstrated that the heaviest retrovirally detected innervation of the VTA by the LDT sourced from glutamate-containing cells. Physiological studies revealed that stimulation of the LDT resulted in glutamate receptor antagonist sensitive excitation of DA neurons [73]. When examined in light of the findings from the CPP study, these data suggested that glutamate input from the LDT was important in LDT activation-dependent CPP, and therefore, by extension, glutamate input from the LDT might be important in drug-induced CPP. Further support of a role played by the glutamate projections from the LDT in drug addiction physiology is provided from findings that AMPA injection into the LDT of chronically amphetamine-exposed rats resulted in higher levels of DA efflux in the nAcc which was paralleled by a greater glutamate efflux within the VTA [72]. However, several findings have lead to the suggestion that the glutamate projection from the LDT to the VTA, while involved, does not play a key role in control of the LDT of burst firing of DA neurons necessary for the large efflux of DA within the mesoaccumbal pathways associated with development of drug dependency. Efflux of DA within the nAcc elicited by stimulation of the LDT was blocked if muscarinic and nicotinic cholinergic receptor inhibitors were present in the VTA suggesting a major role of ACh in control of DA VTA cell firing [174]. Glutamate microinfusion within the VTA of LDT-inactivated animals, while it did significantly increase firing rate of DA cells, it failed to restore burst firing of DA neurons [87]. Accordingly, taken together, some findings suggest a key role of glutamate projections sourcing from the LDT in DA efflux and development of drug addiction-associated behaviors, whereas other studies suggest that glutamatergic input from the LDT is not sufficient to induce behaviorally relevant firing within the VTA necessary for development of these behaviors. Optogenetic methods combined with exposure to drugs of abuse may be used in future studies to more selectively inhibit or activate subpopulations of glutamate or cholinergic LDT neurons in conjunction with the stimulus of drug exposure in order to more fully elucidate the respective roles of the cholinergic and the glutamatergic population of VTA-projecting LDT neurons in DA VTA neuronal firing and development and maintenance of drug addiction-associated behaviors. 

At this time, the role that putative GABA-containing terminals projecting from the LDT may play within the VTA in control of mediating DA VTA neuronal responses to behaviorally salient stimuli have not been examined. The projection pattern of these cells [32] does not support the conclusion that this pathway plays a significant role in control of excitability of mesoaccumbal DA cells. However, some studies have provided data suggesting a role of GABAergic LDT neurons in drug addiction-associated mechanisms. Intercranial self-stimulation results in cholinergic receptor antagonist sensitive rises in DA within the VTA [175]. This phenomenon is believed to be mediated by a polysynaptic pathway involving the mesopontine cholinergic neurons including the LDT [176–178]. This stimulation results in cFos activation of GABAergic LDT neurons suggesting a role of these cells in reward-associated mechanisms [179]. Further, nicotine application has been shown to lead to cFos expression in GABAergic LDT neurons indicating a role of activation of GABAergic cells in neurobiological actions of this rewarding drug [180]. However, these cells may not be those that project to the VTA and the role played by these cells may only involve input directed to non-VTA sites which could include innervation of local LDT cells [180]. Certainly, clarification of the participation of GABA-containing LDT afferents in shaping firing patterns or behaviors mediated by the VTA should be a subject of future direct examination. 

## 13. The Role of Input from the mPFC to the LDT

The medial prefrontal cortex (mPFC) is considered a central component of the brain's classic reward pathway and plays a role in addiction-related, VTA-involved behaviors. For example, administration of a dopamine receptor agonist specific to actions at the D1 receptor into the mPFC reinstates cocaine seeking in an animal in which this behavior had been extinguished [107]. Supporting a role for the mPFC in control of addiction-related processes, it has been shown that stimulation of the mPFC results in activation of DA VTA neurons resembling burst firing [181] and enhancement of DA release in the nAcc [182–185]. Disruption of mPFC neuronal functioning by lesion or chemical inactivation resulted in reductions of DA in the mesoaccumbal pathway, indicating that physiological activity of the mPFC could exert a tonic excitatory influence on DA VTA release [182, 184]. Countering this interpretation is the finding that stimulation of the mPFC at frequencies mimicking those occurring naturally during cognitive events resulted in a reduction of DA efflux within the nAcc [186]. However, as subjects in this study were not drug exposed, it could not be ruled out that drug addiction is a nonnatural state in which activation of the mPFC is capable of activating DA release in the VTA. This activation may be dependent, in part, on a pathophysiological state in which drugs of abuse have induced simultaneous activation of other neuronal groups. While the precise role of activity of the mPFC in drug addiction behaviors remains to be fully elucidated, it is clear that the mPFC can exert an effect on DA release from VTA neurons in the mesoaccumbal pathway. However, this effect on DA release is not likely to be solely subserved via direct cellular actions from mPFC inputs onto DA VTA cells as mPFC terminals do not provide much direct synaptic contact with DA VTA mesoaccumbal neurons and instead synapse selectively on GABA-containing VTA neurons in this circuit [187]. This projection pattern rules out a simple, direct excitation of DA VTA cells as underlying the mPFC control of DA VTA cell excitability and other pathways must be involved in the control exerted by the mPFC on DA VTA functioning. The LDT receives a heavy glutamate-containing projection from the mPFC [41, 188], and it has been hypothesized that the mPFC exerts control of VTA DA neuronal activity via a polysynaptic pathway involving glutamate excitation of the LDT. The mechanistic basis underlying the control the mPFC exerts on the VTA is likely complicated. However, if relays via the LDT are involved in this control, glutamatergic neurotransmission could be directed selectively onto distinct populations of noncholinergic and/or cholinergic LDT neurons. Certainly, the ultimate effect the mPFC has on VTA activity and addictive processes, if it includes a polysynaptic pathway involving the LDT in addition to direct pathways to nAcc and non-DA mesoaccumbal VTA cells, must be complex. The final output from a drug-activated polysynaptic pathway would be shaped by many different inputs directed to the LDT and combined with intrinsic drug actions on cells of the LDT. Summation of all these inputs and cellular actions would serve to convolute the output relayed via the LDT. Therefore, deconstruction of the precise contribution of the mPFC to the role played in drug addiction by the LDT via modification of outflow to target regions is a daunting task. However, the mPFC-LDT pathway may exert a significant influence on drug addiction behaviors controlled by the LDT and therefore deconvolution of its role, and the mechanisms involved in influencing LDT outflow to the VTA may reveal a worthwhile pharmacologic target in management of drug dependency. 

## 14. Future Directions

Currently, pharmacologic treatments for drug dependency are largely unsuccessful. In light of the personal and societal toll exerted by this devastating disorder, development of more successful treatments is crucial, and accordingly, research of the neurobiology of addiction represents a high priority. Once recognition of the roles played by the VTA and the nAcc in determination of stimuli salience and processing of reward were recognized, much of the focus of the drug addiction field has necessarily been directed to studies of effects of drugs of abuse on cellular processing within these nuclear structures. As increased levels of DA within the mesoaccumbal pathway are required for inducing motivation and assessing reward of environmental stimuli, DA has long been the neurotransmitter of focus of addiction research. However, not unexpectedly, other neuronal areas and neurotransmitters certainly play an important role in processes underlying addiction and, if mechanistic elucidation of their role is furthered, offer possible antiaddiction treatment targets. A critical mass of data suggests that the LDT may provide one such target and that activity of cholinergic neurons within this nucleus may be altered to modulate the neurobiological experience elicited by exposure to euphoria enhancing drugs with a high addiction liability. The potential offered by the cholinergic cells of the LDT can be functionally explained by strategic connection of the cholinergic arm of this nucleus within two important neural pathways involved in drug-addiction associated behaviors. The LDT is a critical component of the RAS and provides a major innervation of the VTA. The role played by the LDT within these pathways is not mutually exclusive, and likely activity within one circuit is shaped by activity within the other. Drug use and drug-seeking behaviors involve learning processes requiring a high degree of behavioral arousal. Activity of cholinergic LDT neurons, via their connectivity within the RAS is paramount to attention and arousal. Accordingly, limiting their activation upon presentation or administration of arousing stimuli such as drugs of abuse may reduce activity in circuits involved in directing attention to this stimuli. As arousal and attention are critical to processes associated with learning, this strategy could reduce the maladaptive learning leading to continued use of addictive drugs. Naturally, this strategy is risky and could lead to the undesirable side effect of an inability to focus and a generalized reduction in learning, which is maladaptive by itself, and while possibly effective in reducing use of drugs of abuse would not be an antiaddiction strategy likely to be endorsed by society. 

Rather than development of antiaddiction treatment approaches directed at reducing arousal by targeting the LDT's participation in the RAS, a more effective strategy might involve targeting of the subcircuitry within the LDT centrally involved in addiction via connection to the neural areas important for processing of reward and reward-predicting stimuli. In this context, the LDT cells which contribute to the LDT-VTA excitatory pathway represent theoretically appropriate targets. However, viability of this strategy would rely on the ability to selectively target this LDT population. To date, the detection of commonalities of subpopulations of LDT neurons in so far as content of neuroactive substances, firing behavior, intrinsic membrane conductances, and so forth. that comprise the neurons providing the relevant pathway influencing DA VTA neuronal firing underlying drug addiction behaviors has not been possible [32, 73]. However, if drug addiction treatments are to incorporate targeting of the LDT, the success of this strategy may hinge on delineation of relative contributions to drug addiction physiology of subpopulations of LDT neurons. If such a population can be delineated, it might be feasible to selectively target this population so as to reduce LDT cellular activity induced by drugs of abuse or activity leading to promotion of burst firing of DA neurons. At the same time, activity of LDT neurons underlying appetitive and nurturing behaviors necessary for human survival and quality of life must remain intact. Although the role played by the LDT in processing reward to natural stimuli is relatively unknown, the result of a generalized inhibition of the LDT-VTA pathway could be a brain hypoaroused in response to natural, healthy rewarding stimuli. This would be counterproductive in management of drug dependency, as it has been suggested that an individual with a brain hypoaroused is almost certainly left vulnerable to the pleasures of drug use by turning to the use of euphoria generating drugs or other unhealthy behaviors for activation [189]. Accordingly, at this time, a better understanding of which neurons within the LDT are involved in addiction-related behaviors induced by drugs of abuse and whether those cells are similarly involved in processing of natural healthy rewarding stimuli needs to be gained with the hope that it is possible to block selectively drug-induced LDT activity in absence of alteration of activity induced by natural stimuli. Notwithstanding recent data suggesting a role of glutamate LDT input in initiation of a drug addiction-associated behavior, at this time, the preponderance of data suggest that a specific role of the cholinergic circuitry in drug addiction-associated cell firing and behaviors. Further, drugs which show a high addiction liability like nicotine have been shown to directly activate cholinergic LDT cells and indirectly activate them via preferential actions on excitatory afferents. Therefore, one possible avenue to explore is whether locally and transiently blocking high-levels of activation induced by drugs of abuse in the subpopulation of VTA-projecting cholinergic LDT cells result in a reduction in activity of mesoaccumbal DA neurons. If a reduction in activity is detected, efflux levels of DA in target regions could be monitored to determine whether a lower efflux of DA was effectuated and whether in a concordant fashion, drug addiction-associated behaviors were similarly attenuated. Neurons within the LDT are not homogenous in their neurotransmitter content nor, within a phenotype, are they homogenous in intrinsic electrophysiological properties, and differences may emerge from such initial studies that will allow selective and possibly temporary targeting of drug addiction-related cells, while sparing those involved in other neuronal processes or behaviors. In addition, more studies need to be conducted of firing patterns within LDT neurons associated with enhancement of firing with the VTA. The goal of such studies would be identification of differential firing patterns and categorization of firing patterns as those underlying maladaptive behaviors and those underlying behaviors that benefit the individual. The ultimate goal of such identification would be to inform development of strategies for alteration of firing patterns leading to reinforcement of negative behaviors in order to assist in drug abstinence while avoiding abatement of firing underlying reinforcement of positive behavioral outcomes. In the past, studies of the role of the LDT, which has long been believed to be primarily involved in autonomic functions, in the neurobiology underlying behaviors involving cognitive processes such as addiction to drugs of abuse have seemed off the beaten path. However, a body of work conducted over the years, combined with findings from recent studies using novel techniques, has revealed that the neurons comprising the pathway between the LDT and the VTA may represent central players in processes underlying development of drug-addiction behaviors, and accordingly, may provide a viable neural target in the fight against drug dependency.
